# Supplementary material for: The Effects of Silver Sulfadiazine on Methicillin-Resistant Staphylococcus aureus Biofilms
Source: Microorganisms. 2020 Oct 8;8(10):1551. doi: 10.3390/microorganisms8101551 (PMC7600712; doi:10.3390/microorganisms8101551)
Supplement: Supplementary file 1 [file microorganisms-08-01551-s001.pdf]

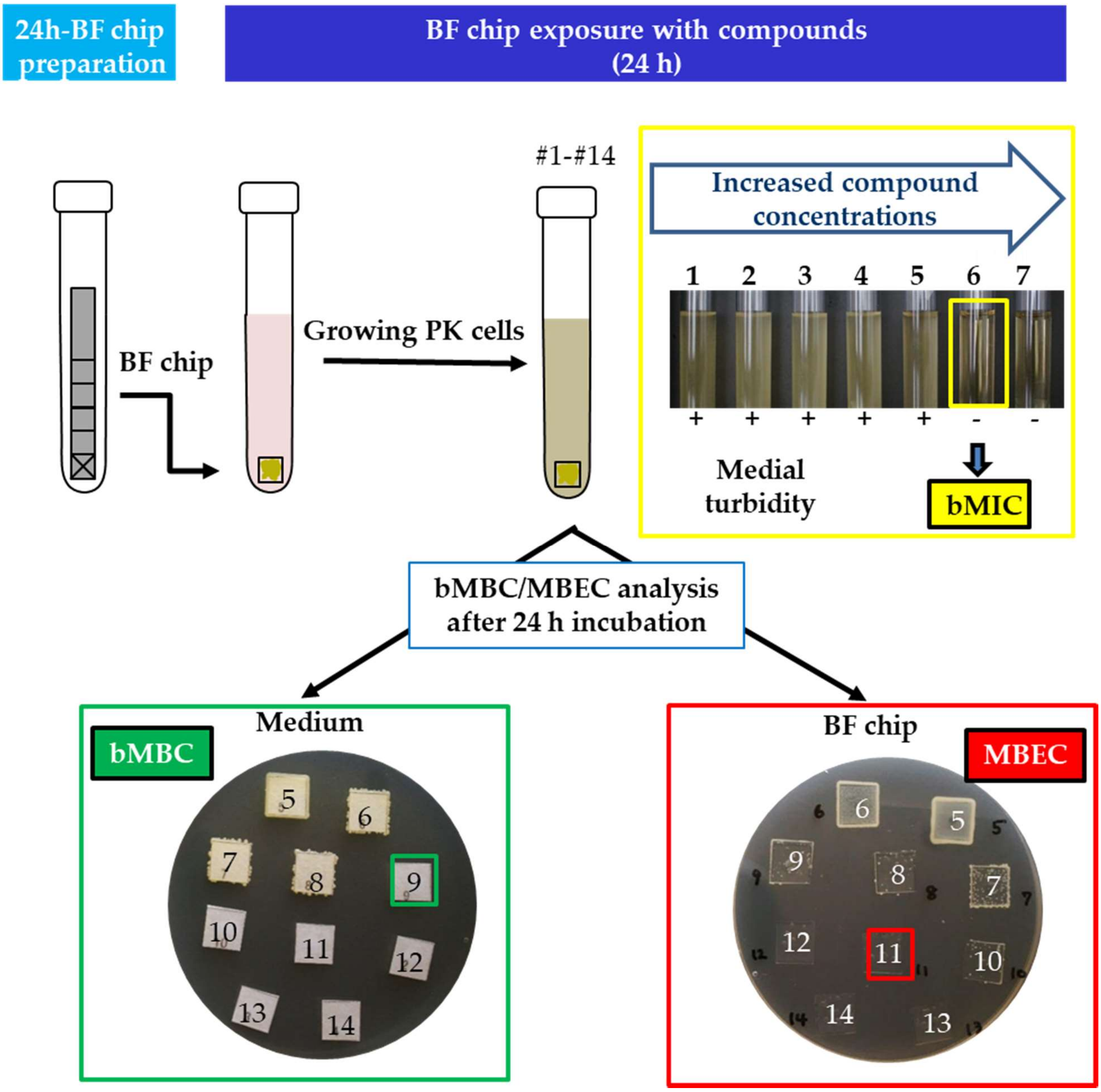

Figure S1. Summarized procedure of the BF chip method to determine bMIC, bMBC, and MBEC.

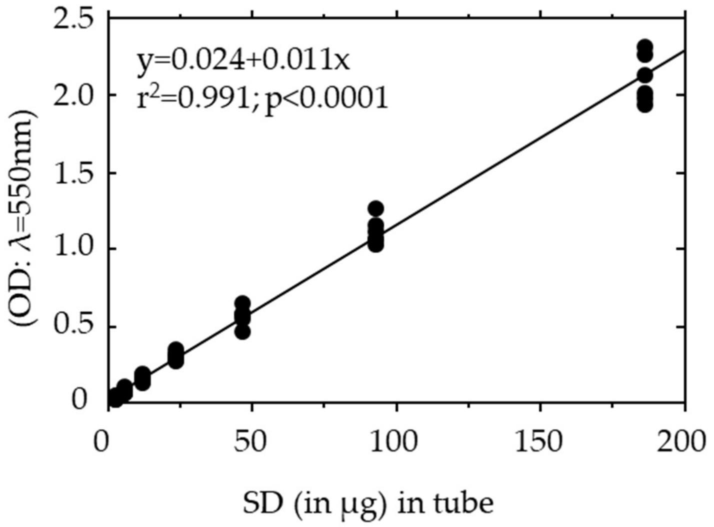

Figure S2. Standard curve of SD.

(a)

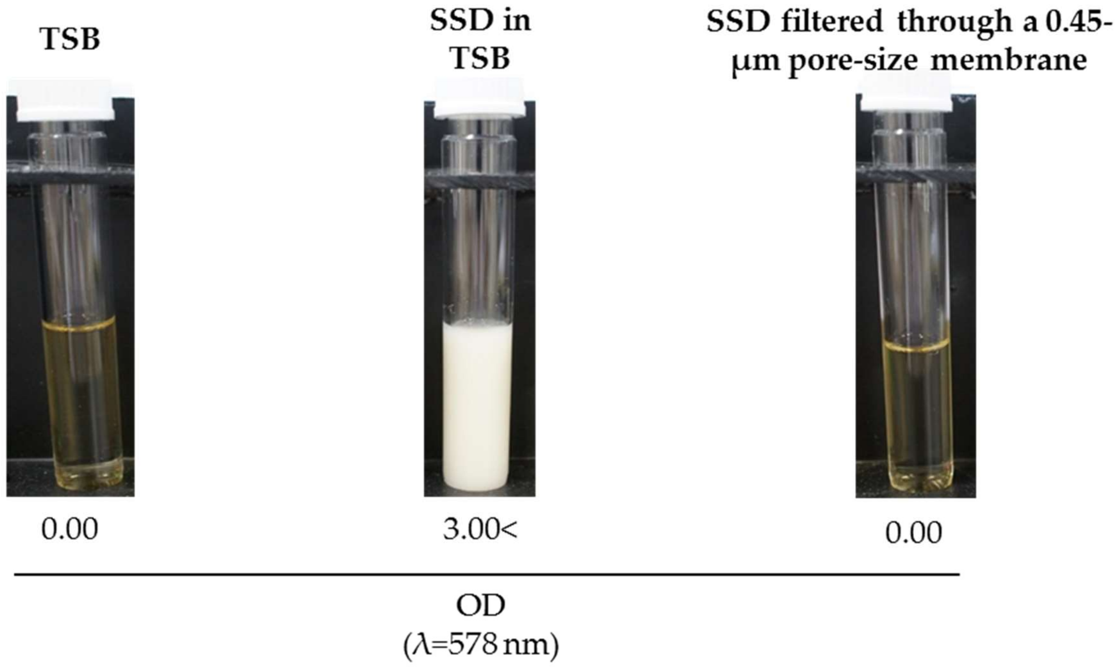

(b)

Group	Silver concentration (μM)							
	0	175	350	700	1400	2800	5600	11200
**AgNO3**	0	0.96 ± 0.14	3.02 ± 0.36	5.91 ± 0.28	7.14 ± 0.28	7.69 ± 0.28	10.03 ± 0.60	18.13 ± 0.48
**SSD (direct)**	0	0.93 ± 0.20	2.27 ± 0.27	4.64 ± 0.31	5.77 ± 0.17	5.25 ± 0.31	6.10 ± 0.20	6.08 ± 0.39
**SSD (chambered)**	0	0.73 ± 0.11	1.41 ± 0.32	1.83 ± 0.57	2.95 ± 0.56	5.23 ± 0.69	5.56 ± 0.64	6.12 ± 0.33

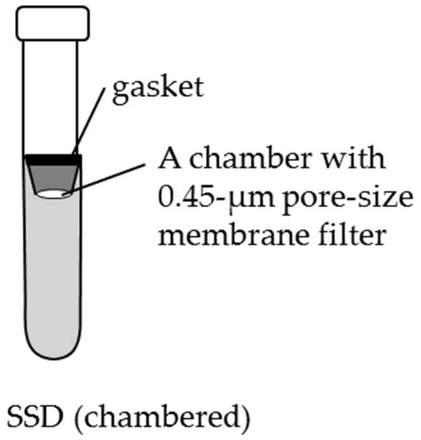

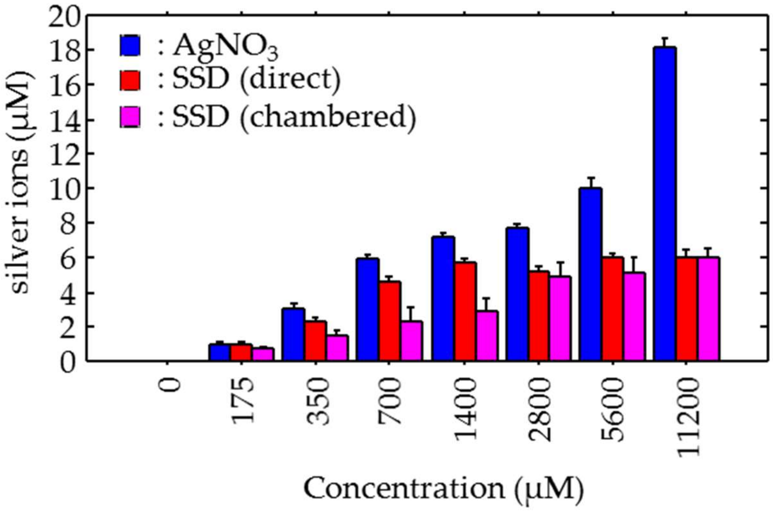

Figure S3. Filtered SSD and ionized silver concentration. (a): The cloudy solution of SSD solution became clear after filtration with a 0.45 μm-pore membrane. (b): Ionized silver concentrations in TSB after 24 h incubation at 37°C with AgNO3, directly added SSD, and chambered SSD. Data are presented as mean ± SE, N size = 3; number of experimental replicates = 2.
